# Infrarenal Infected Aortic Aneurysm Caused by* Streptococcus pyogenes*

**DOI:** 10.1155/2017/9329504

**Published:** 2017-04-19

**Authors:** Floryn Cherbanyk, Markus Menth, Bernhard Egger, Véronique Erard

**Affiliations:** ^1^Department of General Surgery, HFR Fribourg-Cantonal Hospital, 1708 Fribourg, Switzerland; ^2^Department of Medicine, HFR Fribourg-Cantonal Hospital, 1708 Fribourg, Switzerland

## Abstract

Infectious aneurysm is a rare entity associated with significant morbidity and mortality. Current knowledge on pathogenesis, outcome, diagnosis, management, and follow-up remains debatable. We report the case of a patient with* Streptococcus pyogenes* aneurysm who was successfully treated with a homograft implant and discuss microbiological characteristics, diagnostic methods, and treatment options currently available for this serious disease.

## 1. Introduction

Infectious aneurysm, commonly called “mycotic aneurysm,” refers to any aneurysm that result from bacterial and fungal infections of the arterial wall [1, 2]. Before the antibiotic era and improvements in the field of cardiac surgery, infectious aneurysms were usually secondary to infectious endocarditis. Nowadays, atherosclerosis predominantly affecting the aorta and iliofemoral arteries (and its determinants: increasing age, smoking, and male gender) and immunosuppression are the principal predisposing factors of the disease [3]. Bacteria including* Staphylococcus aureus*,* Streptococcus pneumonia, Streptococcus viridans*, and various Enterobacteriaceae especially nontyphi* Salmonella* species are the usual pathogens [4]. Given the lack of conclusive signs and symptoms, patients are subjected to various investigative modalities, until a diagnosis is reached. Antibiotics and open surgery are the standard treatment; however, endovascular repair is also an option. To date, there is no consensus on the best approach.

## 2. Case Presentation

A 69-year-old woman was admitted with a 3-week history of recurrent abdominal pain and intermittent fever up to 39°C. First manifestations occurred about 10 days after she had taken care of her grandchildren, who at the time they were being treated for scarlet fever. In her medical history, she had hysterectomy, bilateral adnexectomy, cholecystectomy, appendectomy, and sigmoïdectomy for diverticulitis.

On admission, the clinical status was relevant only by a diffuse painful abdominal palpation and a temperature of 38,5°C. Blood pressure, pulse rate, and saturation were 115/55 mmHg, 89/min, and 96%. Femoral and peripheral pulses were palpable.

**Figure 1:**
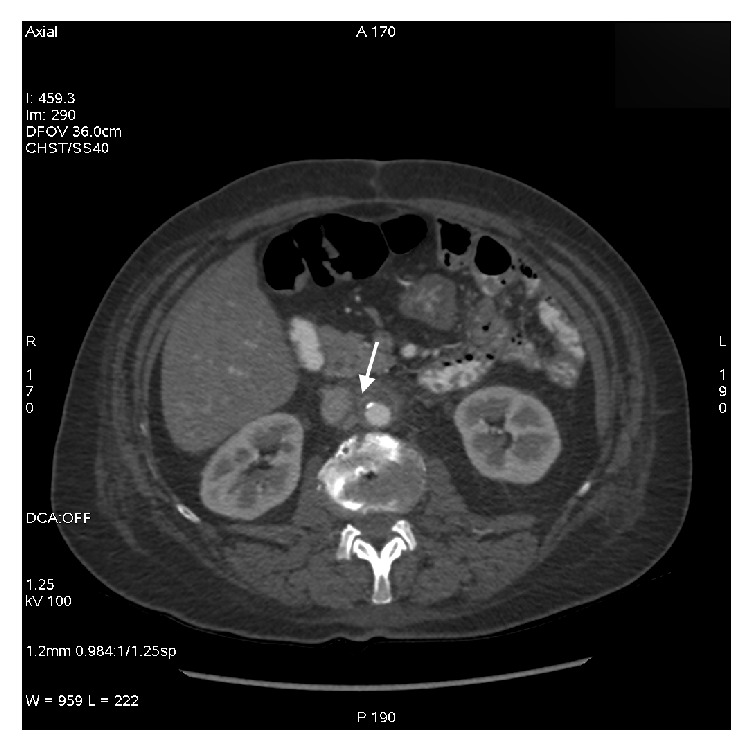
Abdominal CT scan (day 1) revealing infrarenal periaortic infiltrates in contact with the inferior vena cava* (arrow)*.

At admission, C-reactive protein (CRP) was 67 mg/l (normal value <5 mg/l) and leukocyte count was 15.2 G/l (normal value 4.0−10 G/l). Abdominal CT scan showed a circumferential thickening of the sigmoid wall, without fat infiltration, and poorly delimitated infrarenal periaortic infiltrate in contact with the inferior vena cava (Figure 1).

**Figure 2:**
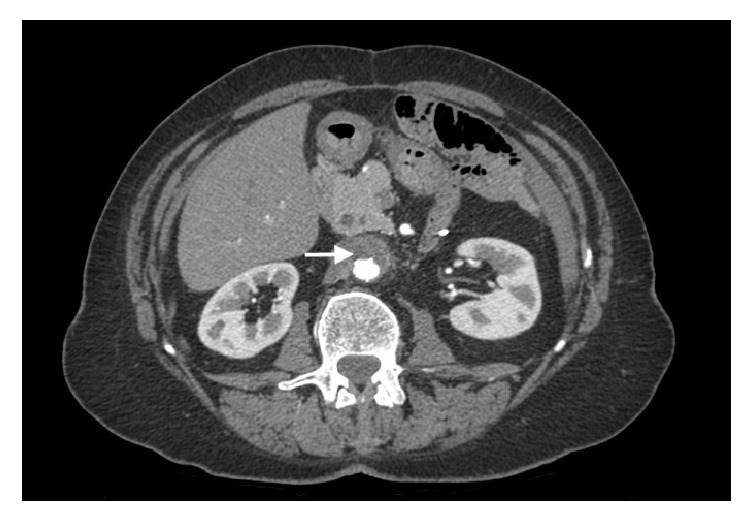
Abdominal CT scan (day 6) showing an increased periaortic infiltration highly suggestive of infectious aortitis (arrow).

A presumptive diagnosis of intestinal infection was made. Piperacillin-tazobactam was initiated. All four blood cultures that were performed were positive for* Streptococcus pyogenes,* causing the initial diagnosis to be reconsidered. A second look of the first CT scan revealed possible signs of an aortic process. A second CT scan on day 6 showed an increased periaortic infiltration highly suggestive of infectious aortitis (Figure 2). Transesophageal echocardiography excluded endocarditis.

**Figure 3:**
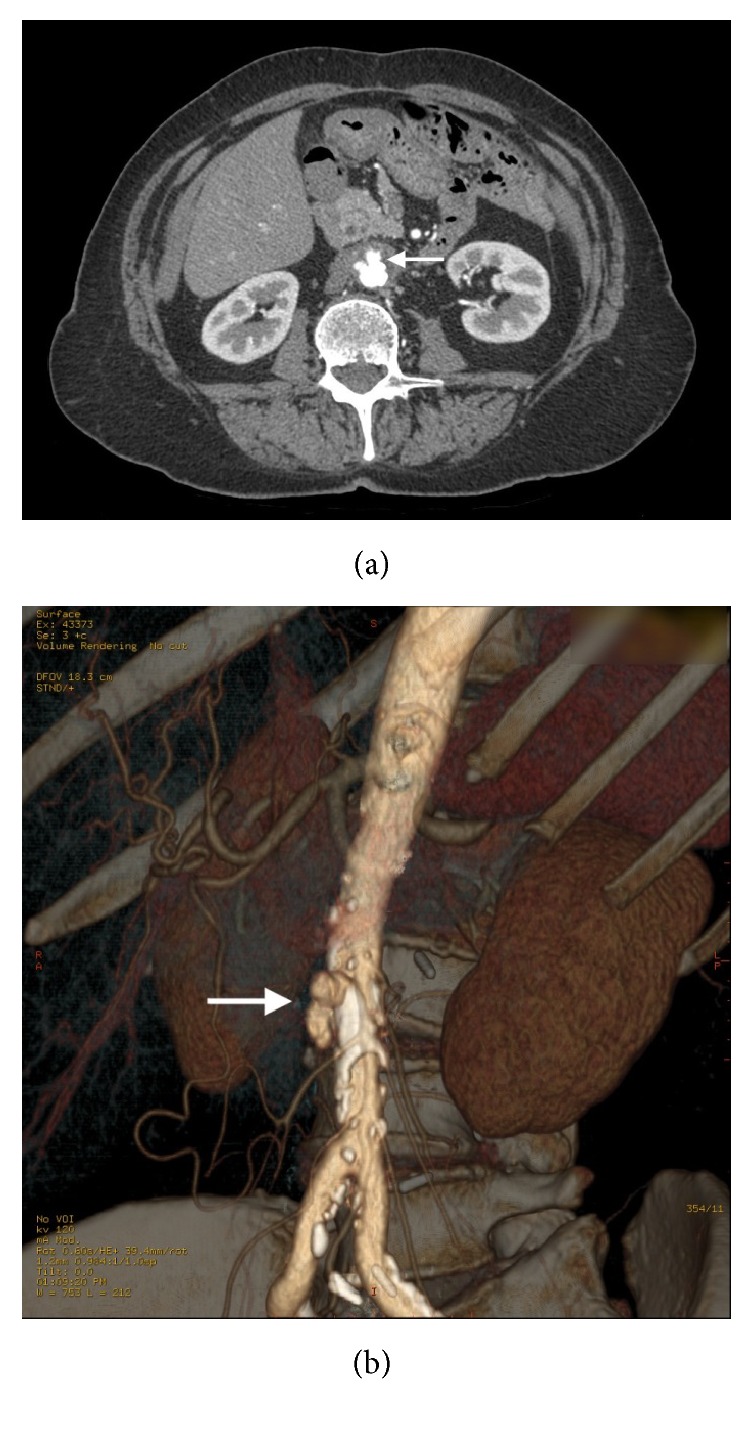
Axial (a) and reconstructed computed tomographic angiography (b) showing signs of prerupture.

**Figure 4:**
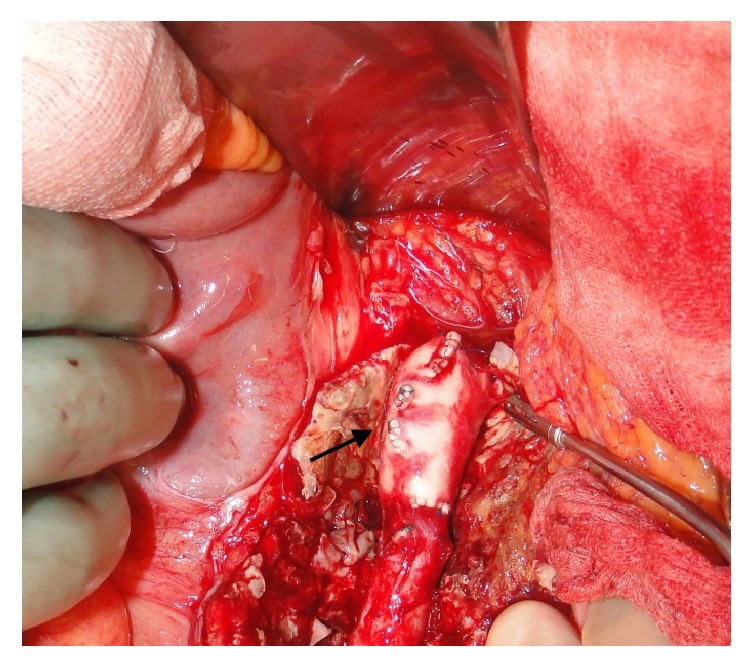
Intraoperative view showing the infrarenal aortic replacement with a homograft* (black arrow)*.

Piperacillin-tazobactam was replaced with ceftriaxone. The patient was referred to a cardiovascular surgery reference center. Vascular surgery was initially not considered. However, one month after admission and despite favorable clinical and biological evolution, the patient experienced severe abdominal pain. The angio-CT scan displayed an infrarenal aortic aneurysm about to rupture (Figures 3(a) and 3(b)), prompting urgent open abdominal surgery. A xiphopubic laparotomy was performed, revealing an infrarenal aneurysm with signs of chronic inflammation. No pus was present externally. After clamping the aorta immediately distally of the left renal artery, as well as the two iliac arteries, the aneurysm was opened longitudinally. The inside contained thrombotic material that was sent for pathological and microbiological examination. The aneurysm was then completely excised and a thoracic homograft was put in place (Figure 4) and covered with porcine pericardium. Cultures of the operative samples remained negative.

Antibiotic therapy with ceftriaxone was continued for two weeks postoperatively and then replaced with oral Clindamycin for 8 additional weeks. The patient made an uneventful recovery and medical and radiological examinations (CT scan) performed at months 6, 12, 24, 72, and 84 after surgery were unremarkable.

## 3. Discussion

Infectious aneurysm is a rare entity, representing less than 2% of all aortic aneurysms [5]. The aorta is normally very resistant to infection, but conditions such as atherosclerosis, malformation, arteriovenous fistula, and bacterial invasion of the vasa vasorum may lead to arteritis and therefore infectious aneurism formation [6–8]. In addition, diabetes, chronic renal failure, chronic steroid exposition, or some degree of immunosuppression can contribute to the development arteritis.

Prior to the antibiotic era, infectious aortitis was commonly associated with bacterial endocarditis involving mostly* S. viridans, S. pneumoniae, *and* Haemophilus influenzae *[9]. Nowadays, the majority of reported organisms seen in infectious aneurysm are staphylococcal species, nonhaemolytic types of Streptococci, and* Salmonella species* [10, 11].* S. pyogenes* is responsible for a diverse range of infectious pathologies but is only exceptionally associated with endovascular infection. In 2013, Gardiner et al. reported one case of* S. pyogenes* aortic aneurysm occurring 4 weeks after an episode of febrile pharyngitis and managed to collect only 8 additional cases from the literature (see Table 1) [12]. In our patient, the only infectious source identified was a prolonged contact with her grandchildren diagnosed with systemic streptococcal infection.

The clinical presentation of infected aortitis is often subtle with a lack of conclusive signs or symptoms. A high index of suspicion is therefore needed in order to reach the diagnosis in the presence of fever, abdominal pain, and/or positive blood cultures of undetermined cause. A significant proportion of blood or tissue cultures remains negative ranging, respectively, from 25–37% and 22–50% [4, 13, 14]. Once the diagnosis is suspected, CT angiography remains the investigative modality of choice, although CT alone or MRI with gadolinium is also adequate in establishing the diagnosis of infectious aneurysm and evaluating the degree of emergency [3, 15, 16].

Infectious aneurysms is still associated with a high mortality rate in spite of advanced antibiotic therapy and improved surgical technic. It is established that the standard treatment of infectious aneurysm is a combination of antibiotics and surgery. To date, there, however, is still no consensus on specific management and the timing and type of surgery as well as antibiotic therapy duration remain debated [17]. Surgical treatment is dependent on numerous factors, including clinical and radiological status, localization of infection, antibiotic treatment prior to surgery, and type of bacteria. Surgery should be delayed if possible, as the outcome for patients with elective surgery seems more favorable than that of patients who require emergency surgery [18].

The standard surgical treatment for infrarenal infectious aneurysm consists of aneurysmectomy, extensive debridement of the surrounding soft tissue, and revascularization [10, 19, 20]. Options for revascularization of infrarenal aorta include in situ reconstruction with use of prosthetic graft or homograft, as well as extra-anatomic bypass and endovascular stent graft [12, 21–23]. The extra-anatomical axillofemoral bypass used to be considered as the standard surgical procedure for infrarenal infectious aneurysms, but outcomes are far from ideal and the magnitude and long duration of the operation compromise its feasibility in severely ill patients. Furthermore, bypass thrombosis is reported in up to 25% of cases despite anticoagulation, and reinfection of the bypass has been reported to be as high as 40% [24, 25]. Finally, hemorrhage of the aortic stump is described in up to 20% of cases [8, 26, 27]. In situ reconstruction with use of a prosthetic graft is attended by a greater risk of graft infection than extra-anatomical bypass procedure. Compared to synthetic grafts, the use of cryopreserved grafts has the advantage of low vulnerability to infection and limited immune response with better viability [28, 29]. This procedure is limited by its cost and the fact that it is not applicable in urgent situations because it needs to be ordered from an international graft banking center. Furthermore, cryopreserved grafts can become dilated on the long term and chronic rejection can lead to local thrombus formation [21]. Reports of open repair of infectious aneurysm indicate a mortality of more than 20% with significant short- and long-term morbidity related to the operation [30], precluding its use in severely ill or shocked patient. Endovascular stent grafts have been introduced as an alternative permitting minimally invasive interventions, prompt aneurysm exclusion, and immediate control of bleeding [31–33]. However, to date, the consensus of expert opinion does not favor the endovascular repair and it is usually reserved for patients with prohibitively high risk for open surgical repair [32]. This restriction is mainly due to the fact that endovascular technique precludes effective drainage of suppuration and debridement of infected tissues. In any case, prosthesis in infected sites can still be used as a temporary treatment prior to definitive open surgical repair [31, 34, 35]. For all these reasons, it is obvious that treatment options need to be evaluated case by case by a multidisciplinary team.

Long-term antibiotics are always indicated; however, their use and duration postoperatively have not been extensively studied [14]. It is reasonable to administer antibiotics for several days or even weeks prior to surgery as long as the clinical situation is stable [36]. In urgent settings, empirical treatment should include large spectrum antibiotics active on Gram-negative Enterobacteriaceae and Gram-positive bacteria, started preoperatively after taking blood cultures. In the absence of positive cultures, molecular diagnostic tests (PCR detection) may help to identify the organisms and determine an adequate postoperative antibiotic treatment. The duration of antibiotic treatment after surgery is not established precisely, ranging from 6 weeks to lifelong therapy [19, 37]. In our case, the length of antibiotic therapy was decided based on the clinical evolution as well as laboratory and radiological results.

Infectious aneurysm is therefore a challenging disease regarding its diagnosis and its management and needs a multidisciplinary plan of treatment.

## 4. Conclusion

Untreated infectious arteritis ultimately leads to aneurysm. Because the clinical signs and symptoms are subtle and unspecific, diagnosis is often delayed until the disease has reached an advanced stage. Elective surgery is preferable to an emergency intervention. Antibiotics are a crucial factor of success and should be initiated as soon as possible; however, there is no universal recommendation regarding postoperative antibiotic treatment. The surgical technique of choice remains disputed, with endovascular repair gaining in popularity but remaining problematic because of graft infection.

## Figures and Tables

**Table 1:** A summary of infectious aortic aneurysms caused by *Streptococcus pyogenes* (table based on that of Gardiner et al.).

Case	References	Year	Age/sex	Site	Surgical treatment	Medical treatment (empiric/directed)	Outcome
1	Valero et al. [38]	1992	65/M	Infrarenal abdominal aorta	Resection with right axillary femorofemoral bypass graft (day 1)	Ampicillin-sulbactam, aztreonam/nafcillin, penicillin G	Died 40 hours after admission because of developed disseminated intravascular coagulation
2	Sing et al. [39]	1994	58/F	Infrarenal abdominal aorta	Aortobifemoral graft	Not discussed antibiotics	Lumbar plexopathy and flaccid paralysis, walking with sticks at 18 months
3	Bisognano et al. [40]	1997	36/M	Aberrant origin right subclavian artery	Ligation of aberrant artery, aortic arch repair with Hemashield graft (day 2)	Not discussed “parenteral antibiotics”	Death day 8: brain death due to diffuse brain ischemia, obstructive hydrocephalus due to cerebellar infarct, transverse sinus thrombosis
4	Barth et al. [41]	2000	1.5/F	Ascending aorta	Aneurysmectomy, valveless aortic homograft (day 8)	Cefuroxime, gentamicin/high dose penicillin G (6 weeks), oral penicillin (3 months)	Well and active at 12 months
5	Chen et al. [42]	2008	81/M	Abdominal aorta	None	Not discussed	Died (aneurysm rupture)
6	Vallejo et al. [43]	2011	72/M	Thoracoabdominal aorta	Open resection, prosthetic graft implantation (rifampicin-impregnated)	Vancomycin, imipenem/penicillin G, benzathine penicillin 1 yr	Well at 6 months on penicillin
7	Leiva et al. [44]	2009	63/F	Thoracoabdominal aorta	Open resection, aortoiliac right, and aortofemoral left bypass with bifurcated prosthetic graft (rifampicin-impregnated) (3 weeks after diagnosis)	Vancomycin, Imipenem/Penicillin G (3 weeks) Penicillin G (1 year) after surgical treatment	Alive at 6 months
8	Hoffman et al. [45]	2012	2/M	Descending thoracic aorta	Open Dacron repair (day 3) and re-operation due to recurrence proximal to original graft	Ceftriaxone, ampicillin-sulbactam/Clindamycin, ampicillin (6 weeks)	Well at 6 weeks
9	Gardiner et al. [12]	2013	60/M	Infrarenal abdominal aorta	Initial endoluminal repair, complicated by secondary graft infection requiring graft excision, axillary-bifemoral bypass	Vancomycin, ceftriaxone, metronidazole/benzylpenicillin (5 weeks), piperacillin-tazobactam (6 weeks), amoxicillin-clavulanate (lifelong)	Well at 4 months, on amoxicillin-clavulanate
10	Present case	2012	69/F	Infrarenal abdominal aorta	Open resection and the infrarenal aorta were replaced with a homograft (4 weeks after diagnosis)	Piperacillin-tazobactam (2 days)/ceftriaxone (4 weeks and then 2 more weeks postoperatively) oral clindamycin (8 weeks) after surgical treatment	Alive at 84 months
